# Prone positioning is associated with increased insulin requirements in mechanically ventilated patients with COVID-19

**DOI:** 10.1038/s41598-024-78904-3

**Published:** 2024-11-12

**Authors:** Harry Griffiths, Amy Cardwell, Max Richardson, Meg Barne, Bogdan Petrisor, Ammara Usman, Laura Heales, Julia Calvo Latorre, Vishakha Bansiya, Razeen Mahroof, Tamas Szakmany, Daniel Martin, Anthony Rostron, Andrew Conway Morris, Sam Lockhart

**Affiliations:** 1 John Farman Intensive Care Unit, Addenbrooke′s Hospital, Cambridge, UK; 2 Grange University Hospital, Cwmbran, Wales UK; 3 Integrated Critical Care Unit, Sunderland Royal Hospital, Sunderland, UK; 4 Department of Anaesthetics & Intensive Care Medicine, Cambridge University Hospitals NHS Foundation Trust, Cambridge, UK; 5 Department of Diabetes & Endocrinology, Cambridge University Hospitals NHS Foundation Trust, Cambridge, UK; 6 Department of Anaesthesia, Intensive Care and Pain Medicine, Cardiff University, Cardiff, UK; 7 Peninsula Medical School, University of Plymouth, John Bull Building, Plymouth, UK; 8 Translational and Clinical Research Institute, Newcastle University, Newcastle Upon Tyne, UK; 9 Division of Anaesthesia, Department of Medicine, University of Cambridge, Cambridge, UK; 10 Division of Immunology, Department of Pathology, University of Cambridge, Cambridge, UK; 11 MRC Metabolic Diseases Unit, Wellcome - MRC Institute of Metabolic Science, University of Cambridge, Cambridge, UK

**Keywords:** COVID-19, Prone ventilation, Glucose metabolism, Insulin, Critical illness, Endocrinology, Endocrine system and metabolic diseases

## Abstract

Stress hyperglycaemia is common in critical illness. We have previously observed that increasing severity of respiratory failure in patients with severe COVID-19 is associated with increased insulin demand. Given previously reported direct effects of hypoxia on insulin action, we reasoned that rapid improvements in oxygenation following prone positioning may improve insulin sensitivity and increase risk of hypoglycaemia. A retrospective multi-centre service evaluation comparing blood glucose and insulin administration in patients with COVID-19 pneumonitis receiving prone mechanical ventilation, comparing the 16 h pre-prone and 16 h post-prone time periods. 155 patients were included in this analysis. Oxygenation improved significantly following prone positioning (change in SpO_2_/FIO_2_ per hour prone: 3.01 ± 0.14, *P* < 0.0001). Glycaemic control was similar during the supine and prone study periods, and there were no hypoglycaemic events in the prone study period. Prone positioning was associated with an unexpected modest but significant increase in insulin requirements (mean difference in total insulin dose (IU): 8.32 ± 2.14, *P* < 0.001) that was robust to several sensitivity analyses, and could not be explained by changes in carbohydrate intake. We did not observe an increased rate of hypoglycaemia during prone ventilation and the adequacy of glycaemic control was comparable during the supine and prone study periods. Unexpectedly, prone ventilation was associated with an increase in insulin requirements despite significant improvement in hypoxaemia. Our findings support the safety of prone ventilation with respect to glycaemic control and identify a novel relationship between ventilation position and insulin requirements in critical illness.

## Introduction

Stress hyperglycaemia is a common complication of critical illness, with approximately 40% of all intensive care unit (ICU) patients requiring insulin administration to control blood glucose^1^. Treatments guided by large randomised studies prioritise the avoidance of hypoglycaemia while minimising severe hyperglycaemia^2,3^.

Patients with COVID-19 frequently demonstrate hyperglycaemia and specific diabetogenic effects of SARS-CoV-2 have been postulated^4^. However, it is unclear how pathogenic features of acute hypoxaemic respiratory failure (AHRF) or its treatments impact glucose homeostasis. In a recent study of glycaemic control in patients with severe COVID-19 and non-COVID-19 viral pneumonias, we observed that increasing severity of respiratory failure was associated with increased insulin requirements^5^. This is intriguing, as evidence exists to support a direct effect of oxygenation on insulin sensitivity. In humans with Type 2 Diabetes, randomised controlled clinical trials have demonstrated that hyperbaric oxygen therapy can rapidly improve insulin sensitivity^6,7^. Conversely, in cell and animal studies, genetic and pharmacological potentiation of Hypoxia-inducible Factor (HIF) signalling enhances insulin action in cells and improves glucose homeostasis in mice^8–11^. However, the relevance of these findings to systemic glucose metabolism in the context of acute illness is unclear.

Prone ventilation is an effective intervention for AHRF, frequently resulting in rapid improvements in oxygenation and improved survival^12,13^. However, the effects of prone positioning on glycaemic control in ICU have not previously been described. Given our observation that severity of respiratory failure was associated with insulin requirements, and the existing data suggesting that increases in oxygenation potentiate insulin sensitivity, we were concerned that improvements in oxygenation and respiratory physiology caused by prone ventilation could result in rapid increases in insulin sensitivity and therefore pose an increased risk of unanticipated hypoglycaemia. Thus, we aimed to determine the safety of prone positioning with respect to glycaemic control, conducting a retrospective multi-centre observational evaluation of glycaemic control and insulin use in mechanically ventilated patients who underwent first-episode prone positioning for AHRF due to severe COVID-19 pneumonitis.

## Results

### Study population characteristics

**Fig. 1:**
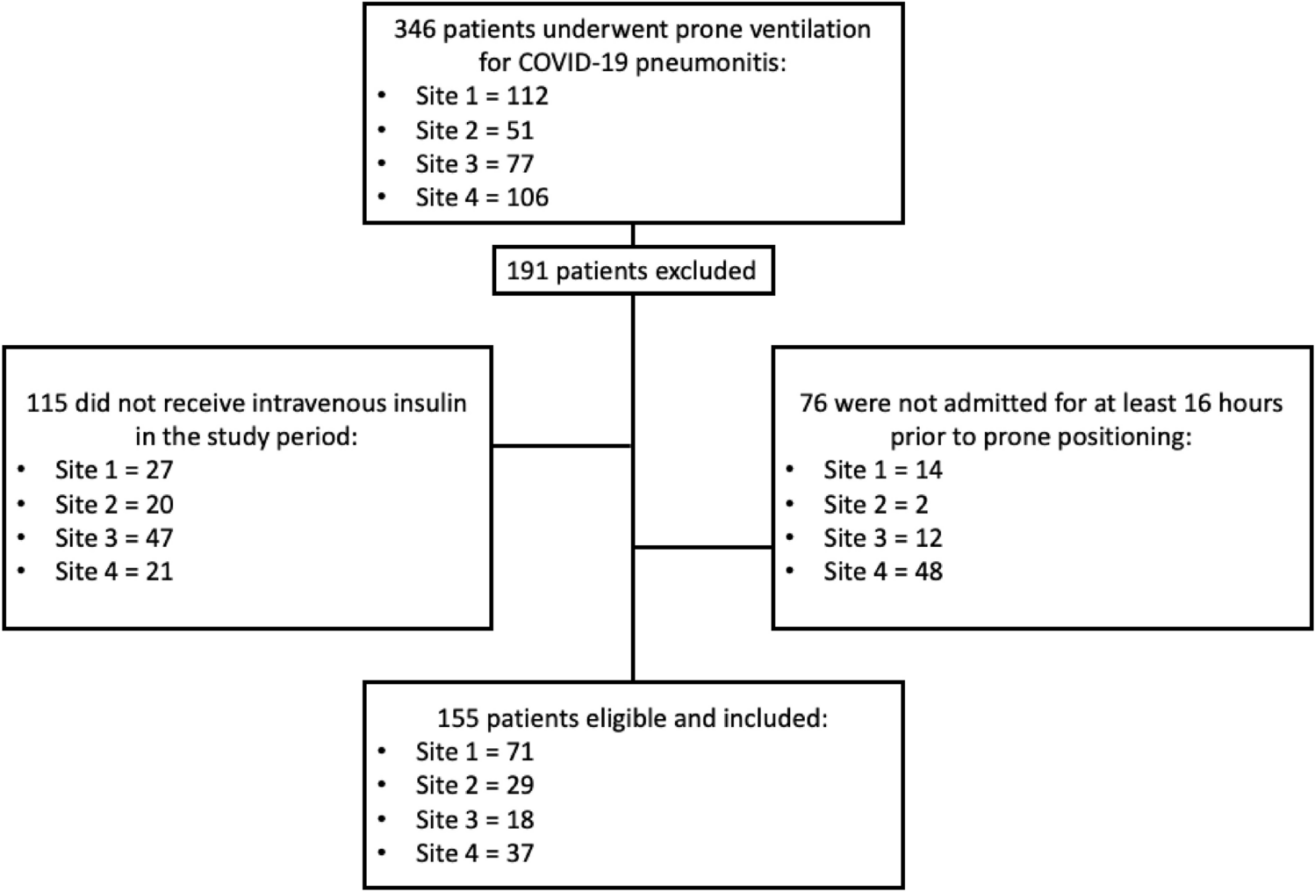
Flow chart detailing patient inclusion/exclusion.

We screened 346 patients who underwent prone ventilation for AHRF due to COVID-19 across all four centres and identified 155 eligible for inclusion. Full details of included and excluded patients are outlined in Fig. 1. The median [interquartile range (IQR)] age of included patients was 61 [52–67]; the majority were male (72.3%), non-diabetic (63.2%), and exposed to corticosteroids during the study period (80.6%) (Table 1).

**Table 1:** Participant characteristics. Continuous variables median(IQR), categorical variables N(%).

	Site 1 (N = 71)	Site 2 (N = 29)	Site 3 (N = 37)	Site 4 (N = 18)	Overall (N = 155)
Age	61.0 (14.0)	63.0 (18.0)	60.0 (12.0)	58.5 (17.0)	61 (15.0)
BMI	31.2 (8.4)	31.1 (9.5)	31.2 (6.7)	30.0 (6.5)	31.2 (8.0)
Sex (M)	51 (71.8%)	25 (86.2%)	29 (78.4%)	7 (38.9%)	112 (72.3%)
Diabetes	21 (29.6%)	8 (27.6%)	15 (40.5%)	13 (72.2%)	57 (36.8%)
Steroid Exposure	62 (87.3%)	20 (69.0%)	29 (78.4%)	14 (77.8%)	125 (80.6%)
APACHE II	14.0 (7.0)	16.0 (8.0)	13.0 (6.0)	16.0 (3.8)	14.0 (7.0)
Days from admission to prone ventilation	5 (6)	5 (4)	3(3)	Not available	5 (5)

### Prone ventilation increases oxygenation but does not increase risk of hypoglycaemia

**Fig. 2:**
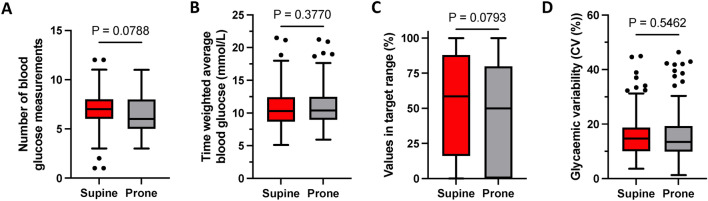
The effect of prone ventilation on glycaemic control in severe COVID-19. (**A**) Tukey boxplot of number of blood glucose measurements in each study period. (**B**) Tukey boxplot of the time weighted average blood glucose in each study period. (**C**) Tukey boxplot summarising the number of blood glucose values in target range (see methods) for each participant during the supine and prone study periods. (**D**) Tukey boxplots displayed as the co-efficient of variation in glucose across the whole study period. A + C: *P* = P-value from Wilcoxon signed rank test, B + D: *P* = P-value from paired t-test. The dots represent values which are greater/less than median ± 1.5 × IQR.

Oxygenation, as assessed by the SpO2/FIO2 ratio, declined during the supine comparator period (Supplementary Fig. 2A, change in SpO_2_/FIO_2_ per hour supine: -2.13 ± 0.14, *P* < 0.0001) and increased following proning (Supplementary Fig. 2A-C, change in SpO_2_/FIO_2_ per hour prone: 3.01 ± 0.14, *P* < 0.0001). No statistically significant difference was seen in the intensity of blood glucose monitoring, time-weighted average blood glucose, blood glucose values in the target range (6–10 mmol/L) or glycaemic variability between the supine and prone periods (Fig. 2A–D). The rate of hypoglycaemia overall was low, with only two biochemically mild (2.8–4.0 mmol/L) hypoglycaemic events occurring during the whole study, both during the supine study period. A minority of participants had any blood glucose values less than 6 mmol/L (proportion of participants with any blood glucose value < 6 mmol/L; supine: 11%, prone: 14%, Fisher’s exact test, *P* = 0.86).

### Prone ventilation is associated with increased insulin requirements in severe COVID-19

**Fig. 3:**
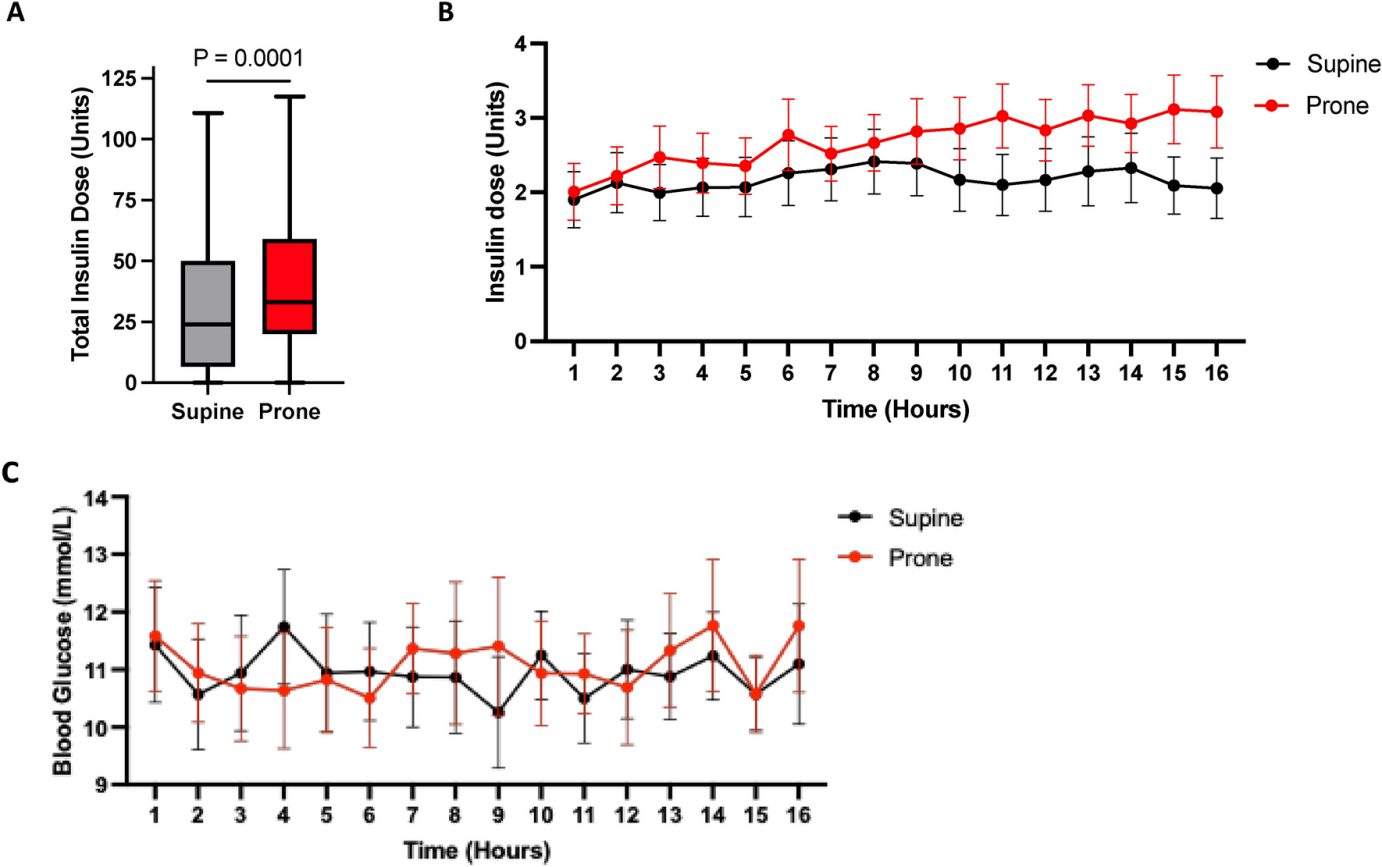
Prone ventilation increases insulin requirements in severe COVID-19. (**A**) Tukey boxplot summarising total insulin doses during the supine and prone study period. P-values from Paired T-Test. (**B**) Graph illustrating the mean ± 95% confidence interval of hourly insulin requirements over time during the supine and prone study periods. (**C**) Graph illustrating the mean ± 95% confidence interval of hourly blood glucose measurements over time during the supine and prone study periods.

**Fig. 4:**
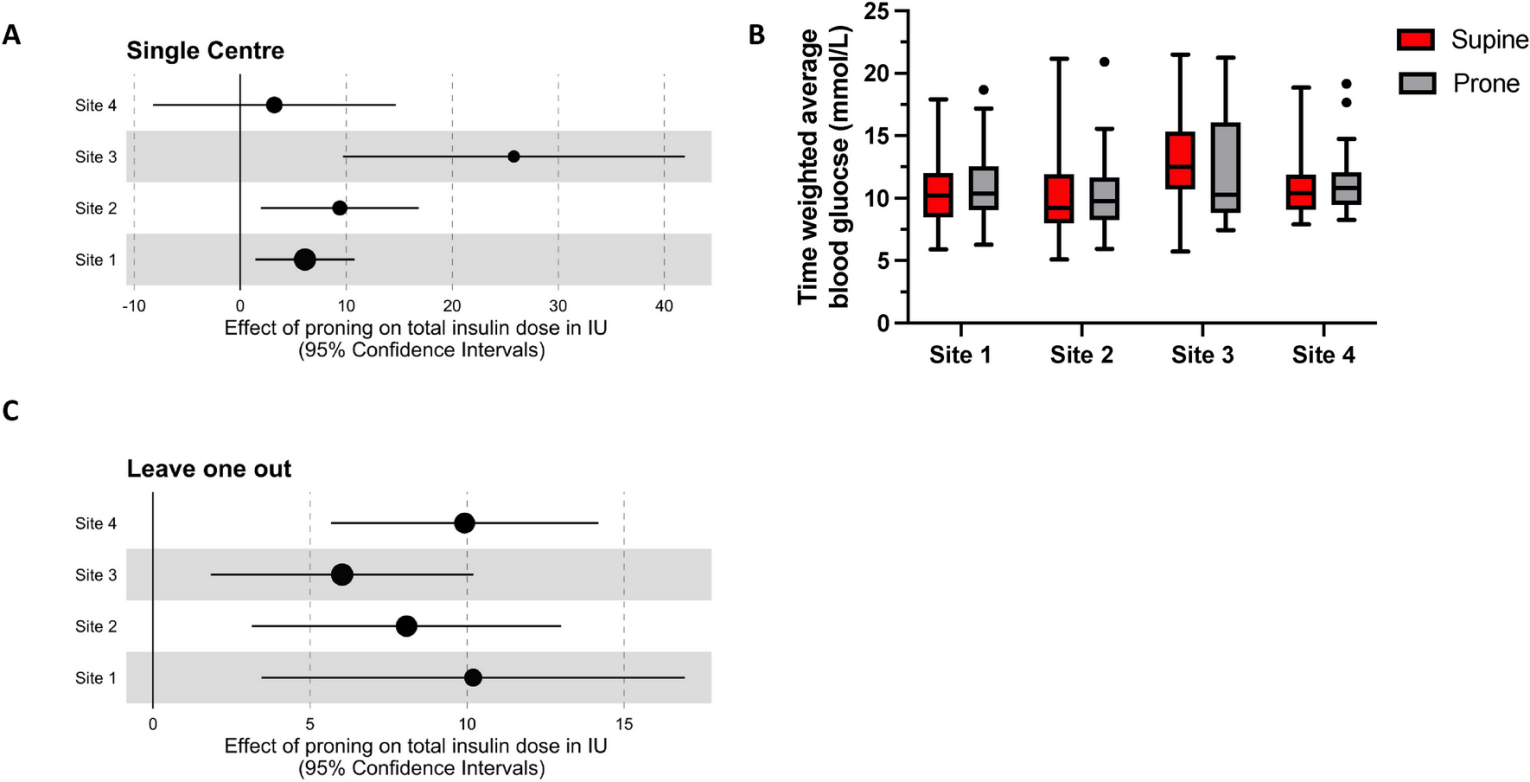
Effect of study centre and time on insulin requirements during prone ventilation. (**A**) Forest plot summarising single-centre analyses illustrating the effect of prone positioning on change in total insulin dose in each study site. (**B**) Box plots illustrating the time weighted average of blood glucose values by centre. (**C**) Forest plot illustrating the effect of removing each study site (indicated on the Y-axis) on the estimate of the change in insulin dose following prone positioning. The effect estimate and 95% confidence intervals in A and C are derived from linear mixed models with each patient having a random intercept.

Prone ventilation was associated with a modest but significant increase in insulin requirements (mean difference in total insulin dose over 16-h periods (IU): 8.32 ± 2.14, *P* < 0.001) (Fig. 3A, B). However, there was no relationship in blood glucose measurements between the supine and prone 16-h periods (Fig. 3C). There was evidence of heterogeneity in the effect size across centres (Fig. 4A), which may be related to differing intensities of blood glucose management during the supine period between centres (Fig. 4B). However, the effect was directionally consistent across all centres (Fig. 4A) and was robust to leave-one-out analysis, where analysis was repeated after removing data from each centre (Fig. 4C).

During the supine period, time was not associated with a change in insulin dose. In contrast, there was a clear time-dependent increase in insulin dose during the prone period (linear regression coefficient ß = 0.07 ± 0.008 units/hour, *P* < 0.0001). Similarly, hurdle regression demonstrated a time-dependent increase in insulin demand during the prone (ß(Hurdle): 0.18 ± 0.02, *P* < 0.0001, ß(linear): 0.006 ± 0.002, *P* = 0.005), but not the supine period (ß(Hurdle): 0.01 ± 0.01, *P* = 0.26, ß(linear): -0.002 ± 0.002, *P* = 0.23 Supplementary Table 2).

### Determinants of increased insulin requirements following prone positioning

**Fig. 5:**
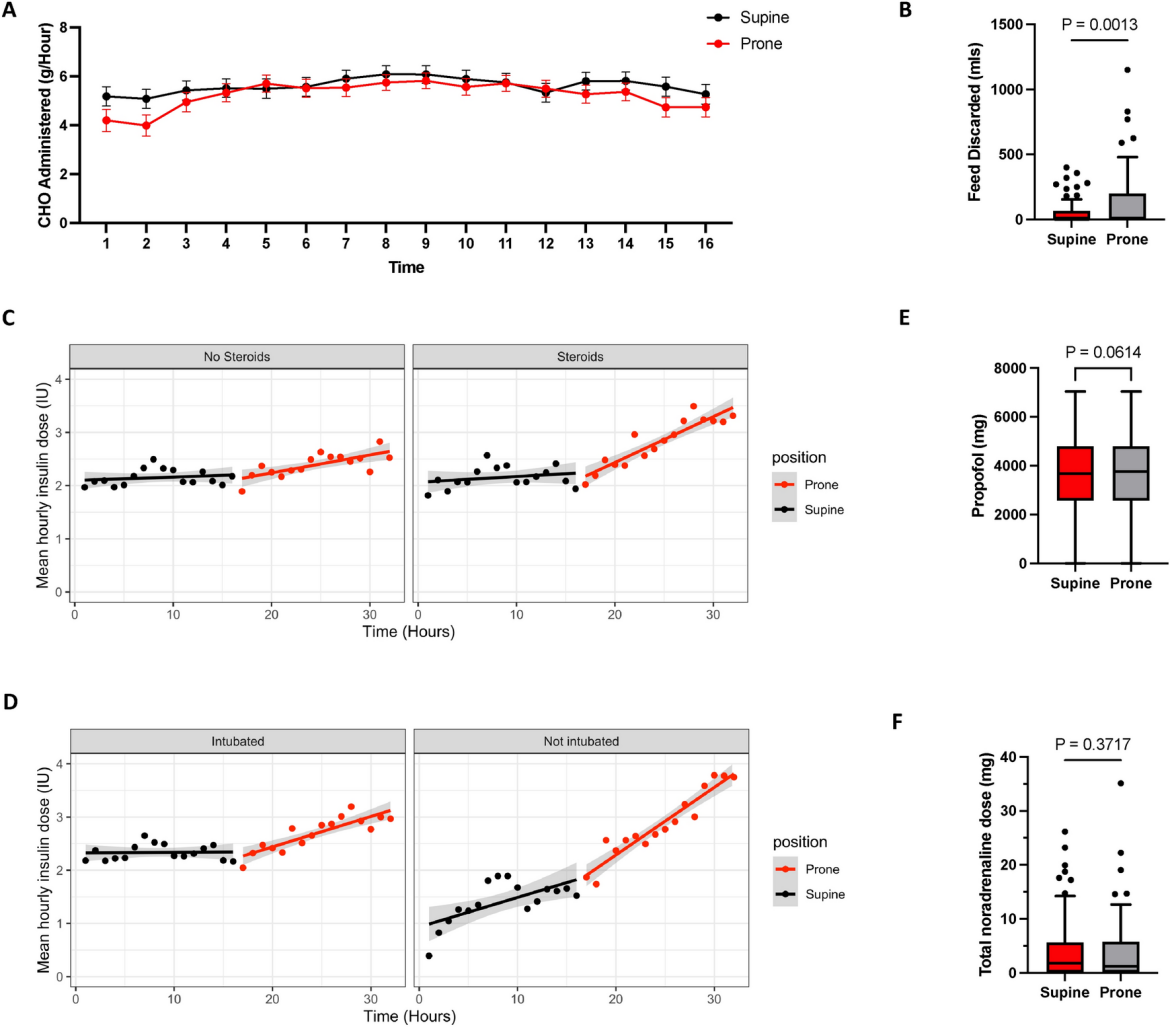
Potential determinants of insulin demand during prone ventilation. (**A**) Mean ± 95% confidence intervals of administered carbohydrate in the supine and prone study. (**B**) Tukey box plot of discarded feed during the supine and prone study periods, P-value from Wilcoxon Signed Rank Test. (**C** + **D**) Scatter plots and regression lines illustrating changes in mean insulin dose over time during the study period in those receiving or not receiving steroids (**C**) during the study period and those who were self-ventilating for a proportion of the supine period (Not intubated) and those who were intubated for the entirety of the study period (**D**). (**E** + **F**) Tukey box plots of Total Propofol (**E**) and noradrenaline dose (**F**), P-value from Wilcoxon Signed Rank Test.

In one centre where we had detailed dietetic data, we found that the administered carbohydrate input was not affected by proning (Fig. 5A), but feed tolerance significantly decreased during proning (median [IQR] volume of feed discarded (ml) prone: 1.5 [0–66.5], supine: 12.0 [0–200], *P* = 0.0013, Fig. 5B). In addition, we observed a significant effect of proning on insulin requirements in a subset of patients who did not receive any steroids during the study period (N = 54) (ß = 0.03 ± 0.008 units/hour, *P* < 0.004, Fig. 5C, Supplementary Tables 1 and 2) with a clear inflection point in insulin requirements upon proning. In addition, in one site with detailed data on steroid administration, steroid use was comparable in both study periods (Supplementary Table 3). We performed a further sensitivity analysis excluding patients who were not mechanically ventilated for the whole supine control period (N = 131 included in the analysis). These results were broadly comparable to the whole cohort: during the supine period, insulin requirements did not significantly increase over time; however, following proning, we observed a progressive increase in insulin demand of comparable magnitude to the estimate for the whole cohort (ß = 0.06 ± 0.009 units/hour, *P* < 0.0001, Fig. 5D, Supplementary Tables 1 and 2), and again an inflection point in insulin requirements was seen upon proning. Neither the difference in AUC FIO_2_ (ß = 0.0008 ± 0.0008, *P* = 0.31) nor AUC SpO_2_ (ß = 0.001 ± 0.002, *P* = 0.48) were associated with a change in insulin requirements between the supine and prone study periods. Propofol and noradrenaline may increase insulin requirements, but doses were comparable in the supine and prone periods (Fig. 5E, F).

## Discussion

We have undertaken, to our knowledge, the first systematic examination of the effects of prone positioning for AHRF on glycaemic control. Our data demonstrate that prone positioning does not meaningfully increase the risk of hypoglycaemia, but does increase insulin demand.

We expected that improvements in respiratory physiology following prone positioning may result in improved insulin sensitivity. Contrary to our expectations, we observed increased insulin demand during the prone study period, an observation that was consistent across included centres and robust to several sensitivity analyses. While this effect was relatively small in magnitude and of uncertain clinical significance itself, it suggests that prone ventilation may have modulatory effects on systemic glucose metabolism. While it has been shown in cells and animals that hypoxia signalling can directly potentiate insulin action^8–11,14^, we could not find any convincing relationship between the severity of hypoxia and insulin doses, arguing against a direct effect of hypoxia and insulin action in this context. It is conceivable that prone ventilation could adversely affect pancreatic perfusion and explain our findings; however, previous studies have not detected an effect of prone positioning on hepatosplanchnic blood flow^15^. While the mechanism underlying our observations remains unclear, it is interesting to speculate that changes in systemic glucose metabolism associated with proning may be linked to its salutatory effects. Our work provides an impetus for more detailed physiological studies on the impact of prone positioning on metabolism.

In a subgroup analysis, carbohydrate intake and feed tolerance decreased during the prone study period and were therefore unlikely to drive the differences in insulin requirements observed. Furthermore, the increase in insulin demand associated with proning persisted in a subset of patients not receiving steroids, and hence this phenomenon was not attributable to steroid exposure. Given the relative consistency of this finding across centres, and the failure of steroids or variation in nutrition to provide an explanation, we considered that this effect could be a direct consequence of the improvement in oxygenation caused by proning, particularly in light of pre-clinical studies suggesting that hypoxic signalling may actually potentiate insulin action^8–11^. However, we found that the change in insulin requirements was not directly related to the changes in SpO_2_ or FIO_2_, thus a biological effect of blood oxygen concentration on insulin requirements seems an unlikely explanation for our observations.

While there was no significant difference in adequacy of glucose control between the supine and prone periods, in almost 50% of patients, half of the blood glucose values recorded during the study period were above the target range. A recent survey of glycaemic control in ICU patients demonstrated that ~ 37% of blood glucose readings in patients who received insulin were above 10 mmol/L despite almost all units surveyed having written glycaemic management protocols^16^. Together, these findings highlight how challenging glycaemic control is in this severely ill population and that routinely implemented glycaemic control strategies are limited in effectiveness. Furthermore, pressures during the pandemic led to deployment of staff with less or no critical care experience and increased patient-to-staff ratios that may have worsened adherence to glycaemic control protocols. Although intensive glycaemic control regimens targeting blood sugars below 6 mmol/L cause harm in the intensive care unit, the tolerable upper limit of target glucose in the critically ill remains unclear^17^. As such, in the absence of the development of hyperglycaemic crises or other immediate complications of hyperglycaemia, it is hard to determine the harm attributable to the burden of hyperglycaemia observed in this patient group.

Our study is limited by its observational nature. In particular, we cannot exclude that treatments commonly implemented alongside prone positioning drive the observed changes in insulin demand. Nor can we exclude confounding by indication, where prone ventilation follows an acute deterioration in patient condition, which itself drives insulin requirements. As such, these findings should be considered hypothesis-generating and worthy of interrogation in further studies. Moreover, we acknowledge the limitations of using gastric residual volumes as a proxy of feed intolerance, which may be influenced by the underlying condition of the patient, the diameter of their feeding tube, and aspiration technique^18^.

In conclusion, we show that prone positioning is safe with respect to glycaemic control and demonstrate an intriguing association between prone ventilation and insulin demand in patients with AHRF due to COVID-19. Healthcare workers should be mindful of increased insulin requirements following prone positioning in critically ill patients, where hyperglycaemia may be detrimental if not monitored attentively.

## Methods

### Study design

We performed a retrospective multi-centre observational evaluation of glycaemic control and insulin use in mechanically ventilated patients who underwent first-episode prone positioning for AHRF due to severe COVID-19 pneumonitis between March 2020 and September 2021 in four centres in the UK. To examine the impact of proning on glycaemic control in patients with severe COVID-19, we collected data on oxygenation (oxygen saturation (SpO_2_ (%)) and fraction of inspired oxygen (FIO_2_)), blood glucose (mmol/L) and insulin dosing (IU) 16 h after the first episode of proning and during the preceding 16 h, which was designated as the supine comparator period (see Supplementary Fig. 1 for the schema of the study design).

### Participating centres

Four centres from different regions of the United Kingdom participated in this study. Cambridge University Hospitals (Cambridge, England (Site 1)) and University Hospitals Plymouth (Plymouth, England (Site 2)) are large teaching hospitals; Sunderland Royal Hospital (Sunderland, England (Site 3)) and Grange University Hospital (Cwmbran, Wales (Site 4)) are district general hospitals. Cambridge University Hospitals included patients meeting inclusion criteria cared for in one of two formal ICUs and surge capacity ICU beds.

### Blood glucose at participating centres

Sites 1 and 3 adopted a blood glucose target of 6–10 mmol/L, while sites 2 and 4 utilised a target of 6–12 mmol/L. Sites 2 and 4 developed guidelines for reduced, standard and increased insulin protocols depending on select patient criteria; site 2 modified these depending on the continuation status of long-acting insulin, as this was continued for patients who were diabetic before admission. All sites recommended the referral of a diabetes specialist team in the event of severe hyperglycaemia. Each of the four sites established a hypoglycaemia threshold of < 4 mmol/L. The standard prescription for hypoglycaemia was 20% glucose 100 ml unless the patient could tolerate oral intake, in which Glucogel 25 g was typically advised.

### Eligibility criteria

Patients were deemed eligible for inclusion if they underwent prone ventilation during their intensive care stay, were present in intensive care for at least 16 h before proning and received intravenous insulin therapy during the 16 h before (supine period) or after (prone period) the first episode of prone positioning. Patients required a positive COVID-19 polymerase chain reaction (PCR) test for inclusion. Patients who received treatment for hyperglycaemic crises during the study period were also excluded. The Cambridge centre screened patients from the inception of the pandemic until February 2021. Sunderland Royal and Grange University Hospital recruited patients treated during 2020. University Hospitals Plymouth screened patients admitted between March 2020 and September 2021.

### Data collection

Relevant, routinely collected clinical data were manually extracted from patient electronic medical records by trained clinical investigators. Demographics and covariates including age, sex, body mass index (BMI), history of diabetes mellitus, steroid exposure, APACHE II (acute physiology and chronic health evaluation) score, were recorded. Hourly measurements of oxygen saturation (SpO_2_), fraction of inspired oxygen (FIO_2_), blood glucose (mmol/L) and administered insulin (IU) were collected from all patients included in the study. Administered insulin was recorded from intravenous infusion records. We also collected enteral feeding data in patients cared for in Cambridge University Hospitals, including carbohydrate intake (g/hour) and volume of feed discarded (ml). In this cohort, volumes of feed discarded were collected 4-hourly while prone and 6-hourly while supine.

### Sample size

All eligible participants screened at participating centres admitted between March 2020 and September 2021 were included. University Hospitals Plymouth screened consecutive admissions from the inception of the pandemic until they had identified 29 eligible participants. As a service evaluation, we did not perform a formal power calculation nor define an overall target sample size.

### Statistical analysis

The effect of prone positioning and time on FIO_2_, SpO_2_ and carbohydrate intake was modelled using linear mixed models with time and position as fixed effects and each patient was allowed to have a random intercept. If a significant interaction was observed between time and ventilation position the linear trend during the prone and supine position were derived and compared using the *emtrends* function in the R package *emmeans*. Adequacy of glycaemic control was summarised using i) time-weighted average glucose calculated by dividing the area under curve of blood glucose measurements by the total time period with available blood glucose measurements in each study period and ii) the percentage of values in the target range as per each centres local protocol. Intensity of monitoring was assessed by comparing the number of blood glucose measurements during each period. Glycaemic variability was reflected using the co-efficient of variation for each participant in each study period, where the standard deviation of blood glucose for each participant in each study period was expressed as a percentage of the mean blood glucose. Difference in feed tolerance was assessed by comparing the total amount of feed discarded in each study period. The insulin administration data contained a high number of zeros, meaning simple linear mixed models with time as a covariate were not appropriate. Therefore, we applied a number of orthogonal statistical approaches to test the effect of proning on insulin requirements; (i) we compared the total insulin dose delivered in each period (ii) we used linear regression to model the relationship between average insulin dose, time and position. Significant interactions were analysed using *emmeans* as described above for FIO2 and SpO2 (iii) we implemented a hurdle regression model with random intercepts using the GLMMAdaptive Package in R. Briefly, this estimates the probability that someone will receive any insulin at a given time point (hurdle part of the model) and the effect of time and position on insulin dose if they receive insulin (linear part of the model). If a significant interaction was observed, the supine and prone study periods were modelled separately. Subgroup analyses to determine the drivers of changes in insulin requirements were conducted using similar approaches. To assess if the change in insulin requirements was related to changes in FIO2 or SpO_2_ by proning we calculated the difference in the area under the curve for FIO2 and SpO_2_ using the *AUC* function in the R package *MESS*. We then regressed the difference in the area under the curve (AUC) for each parameter between the supine and prone periods against the difference in total insulin dose between each study period. The intervals described in the text represent standard errors of the mean unless otherwise stated. Statistical analyses were conducted in R (v4.2.2) (https://www.r-project.org) and Graphpad prism. Between supine/prine period comparisons were undertaken using a paired T-test or, if residuals were clearly not normally distributed, Wilcoxon-Signed Rank test. Visualisation was conducted using R and graphpad Prism.

### Clinical governance and oversight

This study protocol was reviewed and approved as a multisite service evaluation at the host institution (Cambridge University Hospitals Foundation Trust—Service development and Audit Department) and participation of additional centres was confirmed with their respective Audit Departments; hence, the requirement for formal review and ethical approval by an external research ethics committee was waived by the lead organisation (Cambridge University Hospitals Foundation Trust), consistent with National Health Service (NHS) guidelines and regulations for service evaluation. Consent to participate from patients was deemed unnecessary as the data included in this service evaluation were routinely collected and anonymised. This study was conducted in accordance with National Health Service (NHS) guidelines and regulations for service evaluation including registration at the lead organisation and at each site.

## Supplementary Information

Supplementary Information.

## Data Availability

This work is performed as part of a registered service evaluation of routinely collected clinical data and is unable to be shared freely. Data necessary to replicate results reported in this paper may be made available upon reasonable request, subject to approval by participating institutions.

## Abbreviations

ICU: Intensive care unit
AHRF: Acute hypoxaemic respiratory failure
SpO_2_: Oxygen saturation
FIO_2_: Fraction of inspired oxygen
BMI: Body mass index
APACHE: Acute physiology and chronic health evaluation
AUC: Area under the curve
IQR: Interquartile range
