# Supplementary material for: Prone positioning is associated with increased insulin requirements in mechanically ventilated patients with COVID-19
Source: Sci Rep. 2024 Nov 12;14:27668. doi: 10.1038/s41598-024-78904-3 (PMC11557589; doi:10.1038/s41598-024-78904-3)
Supplement: Supplementary file 1 — Supplementary Information. [file 41598_2024_78904_MOESM1_ESM.docx]

**Supplementary Figure 1:** **Schema illustrating the study period.**

**Supplementary Figure 2.** **Effect of prone ventilation on oxygenation in severe COVID-19.**

Parameters relevant to oxygenation are presented for the first 16 hours of prone ventilation and a comparator 16 hour period immediately before prone positioning. Dots and error bars represent mean +/- 95% confidence intervals. Values during supine ventilation are presented in black, and values during prone ventilation are presented in red, N = 155.

**Supplementary Figure 3.** **Effect of prone ventilation on propofol and noradrenaline requirements in severe COVID-19.**

Propofol and Noradrenaline doses are presented for the first 16 hours of prone ventilation and a comparator 16 hour period immediately before prone positioning. Dots and error bars represent mean +/- 95% confidence intervals. Values during supnine ventilation are presented in black, and values during prone ventilation are presented in red, N=71.

**Supplementary Table 1: Effect of prone positioning and time on insulin treatment modelled using hurdle regression**

| **Variable** | **Subgroup** | **Beta.Hurdle** | **Standard Error.Hurdle** | **Pvalue.Hurdle** | **Beta.Linear** | **Standard Error.Linear** | **Pvalue.Linear** |
| --- | --- | --- | --- | --- | --- | --- | --- |
| Time x Position | Whole cohort | 0.13 | 0.02 | <0.0001 | 0.008 | 0.003 | 0.005 |
| Time | Whole cohort | 0.15 | 0.01 | <0.0001 | 0.007 | 0.002 | 0.0005 |
| Position (Prone) | Whole cohort | -0.35 | 0.16 | 0.02 | -0.03 | 0.03 | 0.29 |
| Time x Position | No steroids | 0.09 | 0.03 | 0.002 | 0.005 | 0.004 | 0.31 |
| Time | No steroids | 0.09 | 0.02 | <0.0001 | 0.0008 | 0.003 | 0.8 |
| Position (Prone) | No steroids | -0.39 | 0.27 | 0.15 | 0.03 | 0.04 | 0.4 |
| Time x Position | Intubated | 0.09 | 0.02 | <0.0001 | 0.01 | 0.003 | 0.0005 |
| Time | Intubated | 0.1 | 0.01 | <0.0001 | 0.007 | 0.002 | 0.002 |
| Position (Prone) | Intubated | -0.47 | 0.17 | 0.007 | 0.02 | 0.03 | 0.53 |

Results from a hurdle regression model of hourly insulin dose ~ Time x Position are presented. Subgroup: If model was ran on i) Whole cohort ii) No steroids - those who did not receive any steroids in the periprone period iii) Intubated - Those who were intubated for the whole control period. The ‘.Hurdle’ and ‘.Linear’ suffixes indicate the part of the model which the coefficient refers. Beta-estimates for hurdle regression refer to the log(OR) of receiving any insulin per unit change in relevant predictor variable, whereas Beta-estimates for the linear model refer to the mean change in insulin dose per unit change in predictor variable. The ‘Time x Position’ rows indicate the co-efficient and P-value for the interaction of time and position. Standard error refers to the standard error if the beta-coefficient.

**Supplementary Table 2: Change in insulin requirements over time in the prone and supine position modelled using hurdle regression**

| **Variable** | **Subgroup** | **Position** | **Beta.Hurdle** | **Standard Error.Hurdle** | **PValue.Hurdle** | **Beta.Linear** | **Standard Error.Linear** | **PValue.Linear** |
| --- | --- | --- | --- | --- | --- | --- | --- | --- |
| Time | Whole cohort | Supine | 0.01 | 0.01 | 0.33 | -0.002 | 0.002 | 0.23 |
| Time | No steroids | Supine | 0.005 | 0.03 | 0.86 | -0.002 | 0.002 | 0.31 |
| Time | Intubated | Supine | 0.006 | 0.02 | 0.68 | -0.005 | 0.002 | 0.03 |
| Time | Whole cohort | Prone | 0.18 | 0.02 | <0.0001 | 0.006 | 0.002 | 0.005 |
| Time | No steroids | Prone | 0.12 | 0.03 | <0.0001 | -0.0004 | 0.003 | 0.9 |
| Time | Intubated | Prone | 0.14 | 0.02 | <0.0001 | 0.006 | 0.0023 | 0.01 |

Results from a hurdle regression model of hourly insulin dose ~ Time in the Supine and Prone period in the whole cohort and various subgroups are presented. Subgroup: If model was ran on i) Whole cohort ii) No steroids - those who did not receive any steroids in the periprone period iii) Intubated - Those who were intubated for the whole control period. The ‘.Hurdle’ and ‘.Linear’ suffixes indicate the part of the model which the coefficient and p-values refers to. Beta-estimates for hurdle regression refer to the log(OR) of receiving any insulin per hour, whereas Beta-estimates for the linear model refer to the mean change in insulin dose per hour. Standard error refers to the standard error if the beta-coefficient.

**Supplementary Table 3: Steroid use in the prone and supine periods at Site 1**

|  | **Supine** | **Prone** |
| --- | --- | --- |
| **Number receiving Dexamethasone (%)** | 22 (31.0%) | 17 (23.9%) |
| **Number receiving Hydrocortisone (%)** | 17 (23.9%) | 17 (23.9%) |
| **Number receiving Prednisolone (%)** | 2 (2.8%) | 0 (0%) |
| **Number receiving Methylprednisolone (%)** | 2 (2.8%) | 1 (1.4%) |
| **Dexamethasone dose (95% CI)** | 2.1 (1.3-2.9) | 1.5 (0.9-2.1) |
| **Hydrocortisone dose (95% CI)** | 56.4 (31.2-81.5) | 34.5 (16.0-51.5) |
| **Prednisolone dose (95% CI)** | 1.4 (-0.6-3.4 ) | 0 |
| **Methlyprednisolone dose (95% CI)** | 21.1 (-9.6-51.8) | 7.0 (-6.7-20.8) |
| **Total steroid dose expressed as mg Hydrocortisone (95% CI)** | 14.1 (4.6-23.5) | 6.9 (2.5-11.2) |

95% CI = 95% confidence interval
